# Molecular detection of *Toxoplasma gondii*, *Neospora caninum* and *Sarcocystis* spp in tissues of *Sus scrofa* slaughtered in southern Brazil

**DOI:** 10.1590/S1984-29612023048

**Published:** 2023-08-11

**Authors:** Bibiana Rodrigues de Freitas, Gilneia da Rosa, Isac Junior Roman, Rodrigo Casquero Cunha, Letícia Trevisan Gressler, Juliana Felipetto Cargnelutti, Fernanda Silveira Flôres Vogel

**Affiliations:** 1 Laboratório de Biologia Molecular Veterinária, Departamento de Medicina Veterinária Preventiva, Universidade Federal de Pelotas - UFPel, Campus Capão do Leão, Pelotas, RS, Brasil; 2 Programa de Pós-Graduação em Medicina Veterinária, Laboratório de Doenças Parasitárias, Departamento de Medicina Veterinária Preventiva, Universidade Federal de Santa Maria - UFSM, Santa Maria, RS, Brasil; 3 Laboratório de Microbiologia e Imunologia Veterinária, Instituto Federal Farroupilha, Campus Frederico Westphalen, Frederico Westphalen, RS, Brasil; 4 Laboratório de Bacteriologia, Departamento de Medicina Veterinária Preventiva, Universidade Federal de Santa Maria - UFSM, Santa Maria, RS, Brasil

**Keywords:** Apicomplexa, neosporosis, PCR-RFLP, 18S rRNA, sarcocystosis, Sus scrofa, toxoplasmosis, Apicomplexa, neosporose, PCR-RFLP, 18S rRNA, sarcocistose, Sus scrofa, toxoplasmose

## Abstract

The aim of this study was to determine the presence of deoxyribonucleic acid (DNA) from *Toxoplasma gondii*, *Sarcocystis* spp. and *Neospora caninum*, in tissues of wild boars slaughtered in southern Brazil. A total of 156 samples were collected from different organs of 25 wild boars, and DNA from at least one of the protozoa investigated was detected in 79 samples. To differentiate between infectious agents, restriction fragment length polymorphism was performed using the restriction enzymes *DdeI* and *HpaII*. For *N. caninum*, conventional PCR was performed with specific primers. The DNA of at least one of the studied pathogens was detected in each animal: 26.58% for *T. gondii*, 68.36% for *Sarcocystis* spp. and 5.06% for *N. caninum*. Coinfection between *T. gondii* and *Sarcocystis* spp. occurred in 14 animals, between *T. gondii* and *N. caninum* in only one male animal, between *Sarcocystis* spp. and *N. caninum* in a female, while co-infection with the three agents was equally observed in only one male animal. Considering the high frequency of detection and its zoonotic risk, especially *T. gondii*, it appears that wild boars can be potential sources of transmission of infectious agents and the adoption of monitoring measures in these populations should be prioritized.

## Resumo

O objetivo deste estudo foi determinar a presença de ácido desoxirribonucléico (DNA) de *Toxoplasma gondii*, *Sarcocystis* spp. e *Neospora caninum*, em tecidos de javalis abatidos no sul do Brasil. Foram coletadas 156 amostras de diferentes órgãos de 25 javalis, sendo detectado o DNA de pelo menos um dos protozoários pesquisados em 79 amostras. Para diferenciar entre os agentes infecciosos, o polimorfismo do comprimento do fragmento de restrição, foi realizado usando-se as enzimas de restrição *DdeI* e *HpaII*. Para *N. caninum*, a PCR convencional foi realizada com "primers" específicos. O DNA de pelo menos um dos patógenos estudados foi detectado em cada animal: 26,58% para *T. gondii*, 68,36% para *Sarcocystis* spp. e 5,06% para *N. caninum*. Coinfecção entre *T. gondii* e *Sarcocystis* spp. ocorreu em 14 animais; entre *T. gondii* e *N. caninum* em apenas um animal macho; entre *Sarcocystis* spp. e *N. caninum* em uma fêmea, enquanto a coinfecção com os três agentes foi observada igualmente em apenas um animal macho. Considerando-se a alta frequência de detecção e seu risco zoonótico, especialmente *T. gondii*, constata-se que os javalis podem ser potenciais fontes de transmissão de agentes infecciosos, e a adoção de medidas de monitoramento nessas populações devem ser priorizadas.

## Introduction

Wild boars (*Sus scrofa*) are omnivorous animals and are among the most widely distributed large mammals in the world. Their population has increased significantly in recent decades (Massei et al., 2015), causing damage to crops, competing with native species, and acting as disease reservoirs for wild and domestic animals and humans (Miller et al., 2017). In Brazil, it is considered an invasive exotic fauna, and in several countries, its sport hunting is authorized as a method of limiting its superabundance (Pedrosa et al., 2015; Gazzonis et al., 2018).

In addition carcasses are not inspected and are exposed to precarious environments at the time of slaughter, which can carry several pathogens, thereby transmitting diseases to humans and animals, since a significant number of emerging zoonoses arise from interactions with wild animals. With that the consumption of meat from these animals is a risk factor for human and animal health, especially if it is consumed in raw or undercooked form (Zanella, 2016).

Among the infectious agents in which wild boars can participate in the cycle are the protozoa of the Phylum Apicomplexa, *Toxoplasma gondii*, *Sarcocystis* spp., and *Neospora caninum*, which form tissue cysts. These are of great importance since *T. gondii* and some species of *Sarcocystis* spp. are zoonotic, and *N. caninum* is one of the main causes of abortion in cattle (Hill & Dubey, 2015; Damriyasa et al., 2004).

*T. gondii* infection is considered one of the most significant foodborne diseases transmitted by meat containing viable cysts, one of the main sources of infection, which can result in ocular, congenital, and even fatal toxoplasmosis in immunosuppressed individuals (Hill & Dubey, 2015; Djokic et al., 2016). In animals including pigs, it leads to abortion and neonatal death, resulting in considerable economic loss (Stelzer et al., 2019).

In humans, infections with *Sarcocystis* spp. are generally restricted to the gastrointestinal tract, and *S. suihominis*, in the case of wild boar, is zoonotic with transmission occurring through the ingestion of muscle *Sarcocystis*, which can cause nausea, stomach pain, vomiting, and diarrhea (Fayer et al., 2015; Gazzonis et al., 2019). Furthermore, humans can become definitive hosts for this pathogen after the accidental ingestion of oocysts. Symptoms, such as fever, myalgia, myositis, cough, bronchospasm, itchy rashes, and subcutaneous nodules, have been reported (Rosenthal, 2021), with the largest outbreaks recorded in Southeast Asia (Agholi et al., 2016; Shahari et al., 2016; Lau et al., 2014).

In swine infected with *S. suihominis* and *S. miescheriana*, lower weight gain, skin purpura, dyspnea, muscle tremors, abortion, and death have been reported, in addition to the formation of microscopic cysts in the muscles, even in animals with subclinical infections. Thus, the zoonotic risk associated with consumption of raw or undercooked meat should not be neglected (Dubey et al., 2015).

Another closely related Apicomplexa protozoan, *Neospora* spp., was originally described as causing neuromuscular disease in dogs but is currently a major cause of neonatal mortality, abortion, and encephalitis in ruminants (Lindsay et al., 1995; Lindsay & Dubey, 2020). Although there is no zoonotic evidence, wild boars, which are omnivorous animals, can act as intermediate hosts by developing cysts with bradyzoites in their muscles that can infect domestic or wild canids and other species of intermediate hosts (Haydett et al., 2021).

Despite the authorization for sport hunting, information about the pathogens that affect wild boars and their unique health risks is still scarce. In this context, the objective of this study was to verify the presence of *Toxoplasma gondii*, *Sarcocystis* spp., and *Neospora caninum* in the tissues of wild boar slaughtered in the western region of the state of Rio Grande do Sul, using PCR, Nested-PCR, and RFLP.

## Materials and Methods

### Animals and sample collection

**Figure 1:**
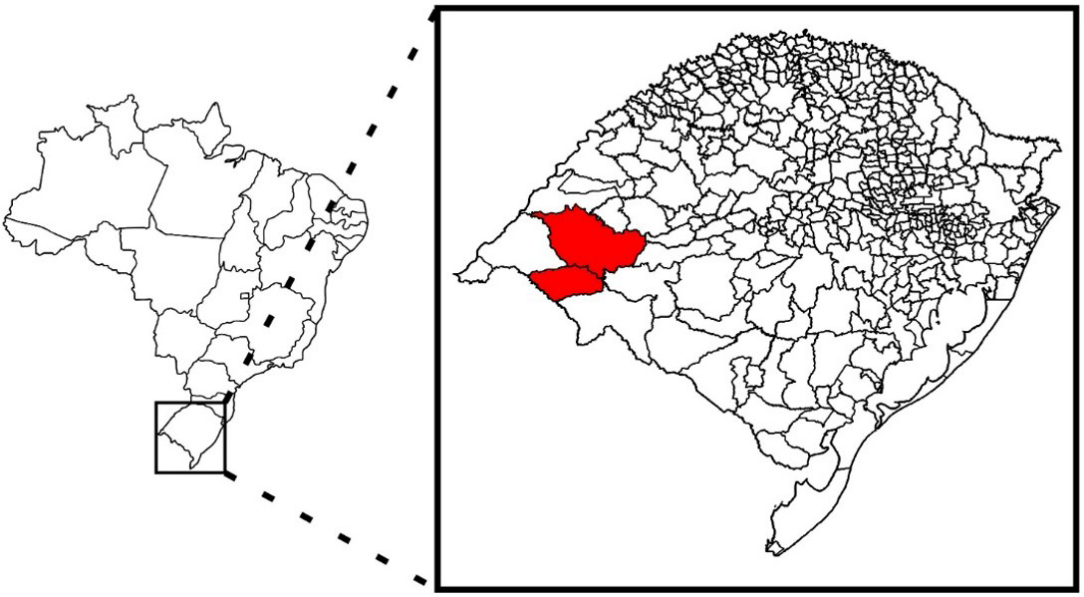
Map of Brazil with the state of Rio Grande do Sul, Brazil highlighted. The western region of the state, where the tissue samples were collected from wild boars hunted for sport, is indicated in red.

A total of 156 *Sus scrofa* tissue samples (15 males and 10 females) were obtained from boars slaughtered through the Official Boar Population Control Program, in accordance with Normative Instruction 03 of January 31, 2013 (Brazilian Institute of the Environment - IBAMA) (Brasil, 2013), in the western region of the state of Rio Grande do Sul, Brazil, as shown in the highlighted area in Figure 1. Tissue samples were collected and stored separately in sterile bags and transported under refrigeration to the Laboratory of Immunology and Veterinary Microbiology of the Instituto Federal Farroupilha (LAMIVET/IFFar). Subsequently, the samples were stored at -20 °C and sent to the Laboratory of Parasitic Diseases of the Federal University of Santa Maria (LADOPAR/UFSM) for molecular analysis. In the laboratory, aliquots of each tissue were collected, namely, the kidney (24), tonsils (21), spleen (25), heart (25), lungs (25), liver (25), diaphragm (3), and testes (8), and subjected to DNA extraction.

### DNA extraction and Nested-PCR

The samples were submitted to DNA extraction using a commercial kit (Wizard® Genomic DNA Purification Kit - Promega®), according to the manufacturer's instructions and with modifications in the lysis step, performed at 55 °C for 16 hours to improve the sarcocyst disruption efficiency according to the protocol adapted by Bräunig et al. (2016). DNA samples were stored at -20 °C until analysis.

After extraction, Nested-PCR reactions were performed with a final volume of 25 µL to detect the agents *Toxoplasma gondii*, *Neospora* spp./*Hammondia hammondi* and *Sarcocystis* spp., through partial amplification of the 18S rRNA gene according to Silva et al. (2009) with adaptations, using, in the first reaction, the external primers Tg18s48F (5'-CCATGCATGTCTAAGTATAAGC-3') and Tg18s359R (5'-GTTACCCGTCACTGCCAC-3') and, in the second reaction, the internal primers Tg18s58F (5'-CTAAGTATAAGCTTTTATACGGC- 3') and Tg18s348R (5'-TGCCACGGTAGTCCAATAC-3'), under the following conditions: 34 cycles with initial denaturation at 94 °C for 5 min, denaturation at 94 °C for 45 s, annealing at 55 °C for 45 s and extension at 72 °C for 45 s, followed by final extension at 72 °C for 5 min in a T100™ Thermal Cycler (Bio-Rad®, USA). The amplified products were visualized on 2% agarose gel in an ultraviolet transilluminator, stained with GelRed Nucleic Acid Stain (Promega®), and the expected final product was 290 bp for *Toxoplasma gondii* and *Neospora spp./ H. hammondi* and 310 bp for *Sarcocystis* spp. *Neospora caninum* strain NC1 and *Toxoplasma gondii* strain RH were used as positive controls, both from cell culture and a previously sequenced positive sample of *Sarcocystis cruzi* and as a negative control, ultrapure water was used.

### Restriction Fragment Length Polymorphism (RFLP)

After the Nested-PCR reactions, the positive samples were submitted to the RFLP technique with restriction enzymes *DdeI* and *HpaII*, according to Silva et al. (2009), in a final reaction of 20 µL, Mili-Q Water 12.6 µL, buffer 2 µL, acetylated BSA 0.2 µL, enzyme 0.2 µL and DNA 5 µL, incubated at 37 °C for 60 min in a T100™ Thermal Cycler (Bio-Rad®, USA). In all reactions, positive controls were used for each researched agent, from previously sequenced samples.

The fragments generated after digestion allowed the identification and differentiation of *T. gondii*, *Neospora* spp./*H. hammondi*, and *Sarcocystis* spp., where *DdeI* digested the *T. gondii* amplicon at two restriction sites, at 182 bp and 110 bp, from *Neospora* spp./*H. hammondi* and *Sarcocystis* spp. at a single restriction site, 290 bp and 320 bp, respectively. For the *HpaII* enzyme, the fragments generated were as follows: *T. gondii* two fragments, 173 bp and 119 bp, *Neospora* spp./*H. hammondi* two restriction sites, 173pb and 120pb, and *Sarcocystis* spp. with a single fragment of 320 bp. The resulting fragments were visualized on a 2% agarose gel and stained with GelRed nucleic acid stain (Invitrogen®), in an ultraviolet transilluminator, according to Figure S1 available in Supplementary Material.

### PCR *Neospora* spp.

Samples positive for *Neospora* spp. and *H. hammondi* were subjected to conventional PCR using primers specific for *Neospora* spp., according to Müller et al. (1996). The reaction was performed using a T100™ thermocycler (Bio-Rad®, USA) and specific primers Np21F (5'-CCCAGTGCGTCCAATCCTGTAAC-3') and Np6R (5'-CTCGCCAGTCAACCTACGTCTTCT-3') in a final volume of 25 µL under the following conditions: 95 °C for 5 min, followed by 35 cycles of 95 °C for 1 min, 60 °C for 1 min, and 72 °C for 1 min; a final extension step at 72 °C for 10 min; and refrigeration at 4 °C, with an expected final product of 328 bp. A sample of *N. caninum* previously sequenced was used as a positive control and ultrapure water as a negative control. The amplified products were visualized on a 1.5% agarose gel stained with GelRed nucleic acid stain (Promega®) in an ultraviolet transilluminator.

### DNA sequencing and analysis

For each species identified in the PCR-RFLP, such as *Sarcocystis* spp., *T. gondii* and *Neospora* spp. a duplicate sample was sent for nucleotide sequencing, from the amplified DNA products of the 18S region. The amplicons were purified using the commercial PureLink ® Quick Gel Extraction Kit and PCR Purification Combo Kit (Invitrogen, Carlsbad, CA, USA), according to the manufacturer's recommendations. Gene sequencing was performed by a specialized company ACTGene - Sequencing Service, Brazil. The samples were sequenced in duplicate, the results obtained were analyzed using the Staden Package software and the similarity with sequences deposited in GenBank determined using the BLAST - Basic Local Alignment Search Tool (NCBI, 2023).

## Results

**Figure 2:**
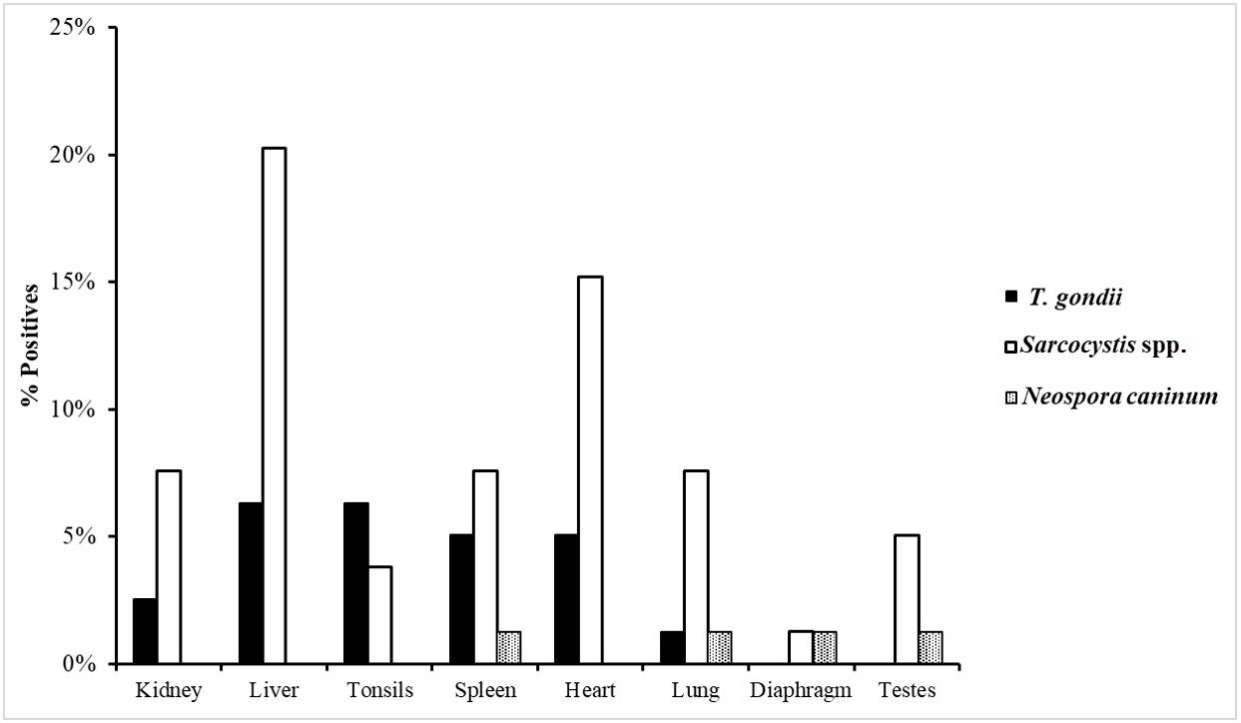
Percentage of tissues positive for DNA of *T. gondii*, *Sarcocystis* spp. and *Neospora caninum*, after Nested-PCR, RFLP and conventional PCR reactions, according to the evaluated tissue of 25 wild boar from southern Brazil.

The results are presented in Table 1 and Figure 2. It was possible to detect at least one of the agents investigated in all animals. Of the 25 animals, only eight did not present coinfection, with six positives for only *Sarcocystis* spp., four females and two males, while only two males were positive for *T. gondii*.

**Table 1:** Molecular detection of *T. gondii*, *Sarcocystis* spp. and *Neospora caninum* in wild boar in southern Brazil according to sex (F: females; M; males) and tissue evaluated. All animals were positive for at least one of the agents.

**TISSUES:**	**KIDNEY**	**LIVER**	**TONSILS**	**SPLEEN**	**HEART**	**LUNG**	**DIAPHRAGM**	**TESTES**
**ANIMAL**								
M1	NC	*Sarcocystis* spp.	NC	NC	*T. gondii*	*Sarcocystis* spp.	NC	NC
M3	*Sarcocystis* spp.	*T. gondii*	NC	*Sarcocystis* spp.	*Sarcocystis* spp.	NC	NC	*Sarcocystis* spp.
M4	NC	NC	NC	NC	*T. gondii*	NC	NC	*Neospora caninum*
M5	NC	NC	NC	*Sarcocystis* spp.	*Sarcocystis* spp.	NC	NC	NC
M6	NC	*Sarcocystis* spp.	NC	*T. gondii*	*Sarcocystis* spp.	*Sarcocystis* spp.	NC	*Sarcocystis* spp.
M7	NC	*T. gondii*	NC	NC	NC	NC	NC	*Sarcocystis* spp.
M8	NC	*Sarcocystis* spp.	NC	*T. gondii*	*Sarcocystis* spp.	*Neospora caninum*	NC	*Sarcocystis* spp.
M9	NC	*Sarcocystis* spp.	*T. gondii*	NC	*Sarcocystis* spp.	NC	NC	NC
M10	NC	*Sarcocystis* spp.	*Sarcocystis* spp.	NC	*Sarcocystis* spp.	NC	NC	NC
M11	NC	*T. gondii*	NC	NC	*Sarcocystis* spp.	NC	NC	NC
M12	*T. gondii*	*Sarcocystis* spp.	NC	*Sarcocystis* spp.	NC	NC	NC	NC
M13	NC	*T. gondii*	*T. gondii*	NC	NC	NC	NC	NC
M14	NC	*T. gondii*	NC	NC	*T. gondii*	NC	NC	NC
M15	*Sarcocystis* spp.	NC	NC	NC	*T. gondii*	NC	NC	NC
M16	NC	*Sarcocystis* spp.	*T. gondii*	NC	*Sarcocystis* spp.	NC	NC	NC
F1	NC	*Sarcocystis* spp.	*Sarcocystis* spp.	*Sarcocystis* spp.	*Sarcocystis* spp.	*Sarcocystis* spp.	NC	NC
F2	*Sarcocystis* spp.	*Sarcocystis* spp.	NC	*Sarcocystis* spp.	*Sarcocystis* spp.	*Sarcocystis* spp.	NC	NC
F3	*Sarcocystis* spp.	*Sarcocystis* spp.	NC	*T. gondii*	*Sarcocystis* spp.	*Sarcocystis* spp.	NC	NC
F4	NC	*Sarcocystis* spp.	NC	NC	*Sarcocystis* spp.	NC	NC	NC
F5	NC	*Sarcocystis* spp.	NC	*Neospora caninum*	NC	NC	*Neospora caninum*	NC
F6	NC	NC	*Sarcocystis* spp.	NC	NC	*T. gondii*	*Sarcocystis* spp.	NC
F7	*Sarcocystis* spp.	*Sarcocystis* spp.	*T. gondii*	*Sarcocystis* spp.	NC	NC	NC	NC
F8	*T. gondii*	*Sarcocystis* spp.	NC	*T. gondii*	NC	NC	NC	NC
F9	NC	*Sarcocystis* spp.	*T. gondii*	NC	NC	*Sarcocystis* spp.	NC	NC
F10	*Sarcocystis* spp.	*Sarcocystis* spp.	NC	NC	NC	NC	NC	NC

NC: not collected.

Two or more agents were detected in 17 of the animals (Table 1). Coinfection with *T. gondii* + *Sarcocystis* spp. in fourteen animals, being nine males and five females; *T. gondii* + *Neospora caninum* in only one male, *Sarcocystis* spp. + *Neospora caninum* in a female and the coinfection with the three agents: *T. gondii* + *Sarcocystis* spp. + *Neospora caninum* in a male animal. The distribution of each pathogen detected according to identification of the animal and tissue collected is shown in Table 1, and the detection rate is shown in Figure 2.

Of the 156 tissue samples evaluated, 79 (50.64%) were positive for at least one of the following agents: 21 (26.58%) for *T. gondii*, 54 (68.36%) for *Sarcocystis* spp., and 4 (5.06%) for *Neospora caninum*. The organs/tissues with the highest frequency of DNA detection, considering their total number in relation to the number of positive samples, were the liver, testis, diaphragm, and heart with 21, 8, 2, and 16 samples, respectively. The organs and tissues with the lowest frequency of detection were the kidney, tonsils, and lung, all with eight samples, followed by the spleen with 11 samples.

All sequenced samples confirm the presence of the three researched agents, with identity of *T. gondii* 99.61-100%, *Neospora caninum* 99.19-99.60% and *Sarcocystis miescheriana* 98.68-99.33% of homology with the partial nucleotide sequences of the 18S rRNA gene deposited in GenBank.

## Discussion

The high frequency of DNA detection in protozoa of the phylum Apicomplexa found in the present study suggests the potential of wild boars as sources of pathogen transmission to both humans and animals. It is of great importance, especially for humans and animals that are associated with wild boar management, not only because of contact with infected tissues and blood but also because of the consumption of raw or undercooked meat. In addition, epidemiologically, these animals are in contact with other wild and domestic animals, which facilitates the spread of these and other unidentified pathogens (Guardone et al., 2022).

A significant percentage of 26.58% of tissues with detection of *T. gondii* corroborates the data from the meta-analysis of toxoplasmosis in wild boars by Rostami et al. (2017), who demonstrated a seroprevalence of 23%, most frequently in North America (32%) and Europe (26%). Lower detection rates were found in South America (5%); however, it should be noted that only two studies with 340 animals were included, and the real prevalence of the infection may be underestimated. In addition, studies on the detection of this agent in wild boars are still scarce and many countries do not present any data on their occurrence (Rostami et al., 2017).

In Brazil, Machado et al. (2019), in a survey in the south and midwest, found a high seroprevalence of *T. gondii* in hunters (32.7%), hunting dogs (31.2%), and wild boars (21.1%), all from the same region, highlighting these wild animals as potential sentinels for toxoplasmosis, especially in anthropized areas. Furthermore, Conrady et al. (2022) described the handling of game carcasses through direct contact with the blood of infected animals and droplet dispersion as a possible source of ocular toxoplasmosis acquired in previously healthy hunters and non-consumers of meat products from wild animals.

Like *T. gondii*, in recent years, the occurrence of *Sarcocystis* spp. in wild boar has been studied mainly by molecular methods in Latvia (Prakas et al., 2020), Italy (Gazzonis et al., 2019), Romania (Imre et al., 2017), Spain (Calero‐Bernal et al., 2016) and Portugal (Coelho et al., 2015), where a prevalence ranging from 8 to 69% of positive animals was found.

However, because of the smaller number of females evaluated in this study it is not possible to infer data on higher or lower frequency of detection in relation to the sex of the animals. Other studies have however, reported male wild boars with higher detection rates for *T. gondii*, ranging from 42% (Bassi et al., 2021) to 60% (Machado et al., 2021). This may be explained by the fact that male wild boars have solitary habits, travel longer distances in search of food when compared to females and young ones, thereby increasing the risk of infection (Machado et al., 2021; Rostami et al., 2017), as oocysts of this pathogen survive for long periods in an aquatic environment (Shapiro et al., 2019). This, combined with the scavenger and predatory habits associated with cannibalism in these animals may be a potential explanation for this observation (Ballari & Barrios‐García, 2014).

In the present study, *Sarcocystis* spp. was the most frequently detected protozoan, with 68.36% and 22 infected animals. This high detection frequency is probably related to the abundance of suitable definitive hosts circulating in the same environment, such as wild and domestic canids, and to the consumption of wild boars by these carnivores; this is the first report of the detection of *Sarcocystis* spp. in wild boars in Brazil.

In swine, in a single report by Espindola et al. (2022), in the central region of the state of Rio Grande do Sul, the frequency of anti-*Sarcocystis* spp. in 36.9% of blood serum samples from domestic animals, using the indirect immunofluorescence technique (IFAT), and a DNA detection rate of 67.9% in tissues by PCR, indicated that there is a wide circulation of the protozoan between animals and properties in the region and the potential risk of infection to humans and domestic animals living in the same place.

Although the zoonotic potential of *Sarcocystis* spp. is known, there are few reported cases of intestinal sarcocystosis in humans due to the consumption of raw or undercooked meat (Rosenthal, 2021). However, clinical infections are believed to be underreported or misdiagnosed because most infections are low-grade and asymptomatic (Poulsen & Stensvold, 2014; Fayer et al., 2015). Eating raw or undercooked wild boar meat can potentially pose a risk to human health.

In addition, according to Rosenthal (2021), hunters play an important role in the spread of zoonotic species of *Sarcocystis* spp., and the supply of the viscera of slaughtered animals to the dogs and remains of carcasses discarded freely in the environment can serve as food and transmit the agent to most of the diverse species that inhabit the place.

Little is known about the exact role of wild boars in the introduction and maintenance of *N. caninum* infection in the wild and its possible transmission to domestic animals (Haydett et al., 2021). In Brazil, studies have demonstrated the presence of antibodies against *N. caninum* in wild boars (Soares et al., 2016), but there are still no reports on the application of molecular diagnostic techniques. Reports of exposure to *N. caninum* in wild boars are limited to the US, with 15% in Oklahoma, New Mexico, and Texas (Bevins et al., 2013) and 15% in 21 states, ranging from 0% in California to 57% in Michigan (Cerqueira-Cézar et al., 2016). In humans, neosporosis is not considered a zoonosis; however, studies have demonstrated the presence of antibodies in hunters and animal handlers who are positive for the agent (Oshiro et al., 2015; Benetti et al., 2009). Furthermore, *N. caninum* DNA has been detected in the umbilical cord of immunosuppressed women, demonstrating its potential for infection in humans (Duarte et al., 2020; Villa et al., 2022).

Although the DNA detection rate was 5.06%, it is important to highlight that wild boars could be intermediate hosts for this agent (Cerqueira-Cézar et al., 2016). These animals may be infected through the ingestion of sporulated oocysts that remain viable in the soil, food, and water for long periods (Haydett et al., 2021).

In domestic swine, the occurrence of *N. caninum* is described in several countries. One of the highest reported seroprevalence cases occurred in Senegal, where 58.3% of the sows were infected (Kamga-Waladjo et al., 2010). In Italy, a prevalence of 6.7% was reported (Villa et al., 2022) and in China, from 0.3% to 4.6%, among animals from different provinces; this being the only study by molecular detection (Gui et al., 2020). In Brazil, the seroprevalence in commercial pigs varies from 3.1% (Azevedo et al., 2010) to 3.2% (Feitosa et al., 2014) in the northeast region, 13.5% (Minetto et al., 2019) in the midwest region, and 18.9% (Silva et al., 2020) in the southern region. According to Villa et al. (2022), domestic pigs are likely to be infected by ingesting water and food contaminated with sporulated oocysts from dogs or tissues with cysts from other intermediate hosts that circulate close to the facilities.

In addition, wild boar carcasses and viscera, as well as meat waste, when not properly disposed of in the home environment, can also act as a source of infection by these agents closely related to new hosts, such as free-living cats, capable of completing the life cycle of *T. gondii* and spread infective oocysts in the environment, and canids, in the case of *N. caninum* and *Sarcocystis* spp., to the most diverse animal species that are scavengers and do not have direct contact with wild animals (Coelho et al., 2015; Mateo‐Tomás et al., 2015).

## Conclusion

Considering that all wild boars showed detection of at least one of the evaluated pathogens, there is a potential risk of transmission of these agents, especially *T. gondii* and *Sarcocystis* spp., through consumption and handling of raw and undercooked meat for both humans and animals. Therefore, it is important that this animal be monitored, as there are a large number of wild boars in Brazil. As the hunting of these animals has increased in recent years, it is also essential to monitor the occurrence of infectious agents in these populations. In addition, all pathogens detected can infect both humans and domestic animals. Therefore, monitoring measures and the correct disposal of carcasses and viscera from wild animals, especially wild boars, should be prioritized.

National Center for Biotechnology Information - NCBI. BLAST: Basic Local Alignment Search Tool [online]. 2023 [cited 2023 jan 10]. Available from: https://blast.ncbi.nlm.nih.gov/

## Supplementary Material

Supplementary material accompanies this paper.

Figure S1 Illustration of the observed pattern of the PCR product (without enzyme), and after digestion by DDEI and HPAII digestion enzymes, in agarose gel.

This material is available as part of the online article from https://doi.org/10.1590/S1984-29612023048
