# Supplementary material for: Molecular detection of Toxoplasma gondii, Neospora caninum and Sarcocystis spp in tissues of Sus scrofa slaughtered in southern Brazil
Source: Rev Bras Parasitol Vet. 2023 Aug 11;32(3):e004623. doi: 10.1590/S1984-29612023048 (PMC10449315; doi:10.1590/S1984-29612023048)
Supplement: Figure S1 [file rbpv-32-3-e004623-Supl.pdf]

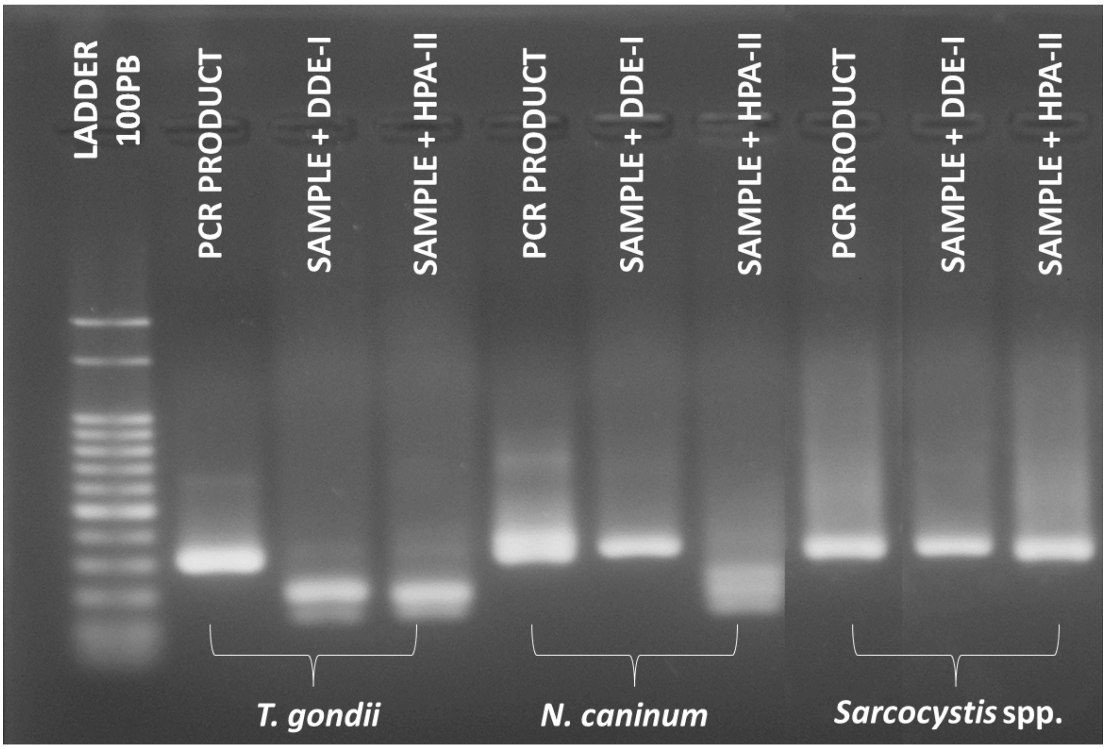

Figure S1: Illustration of the observed pattern of the PCR product (without enzyme), and after digestion by *DDEI* and *HPAII* digestion enzymes, in agarose gel.

* Please do not publish the figure with the manuscript, sent at the request of the reviewer to illustrate the agents' identification.

Sequences obtained from RFLP confirmation – BLAST

Toxoplasma gondii

gaacttgaatgatccgtcgcagaccgaagtcaacgcgatccgttcggttactatgaatcacctgaggaccaccggaaggtgggttggttct
gtatctaataaacactgcccttccaggggaagaggcatgtgcgcatgtattagccatagaattaccacggttatccatgtagtaaagaccatc
aaataaactataactgttttaatgagccattcgcagtttagccgtataaaagcttatacttagacatgcatgga

Sarcocystis miescheriana

tatttgatagtctcaaatactacatggataaccgtggtaattctatggctaatacatgcgcaaatactatatcattctctcncttttgggggtgttg
tgatatagtagtgtttattagatacagaaccaacacatcattattaccatatgaaacttgaatgatctatcgccaatcatcaaataatgataattat
aatgcgatccgttcggttactatgaatcaccttattttacatcaccaccaccataaannatggnaataatgatgtgttggntctgtatctaataaa
cacta

Neospora caninum

ttatttgatggtctttactacatggataaccgtggtaattctatggctaatacatgcgcacatgcctcttcctctggaagggcagtgtttattagat
acagaaccaacccaccttccggtggtcctcgggtgattcatagtaaccgaacggatcgcgtttgacttcggtctgcggcggatcattcaagt
ttctgacctatcagctttcgacggtactgtattggactaccgtggca
